# Visual impairment and falls among older adults and elderly: evidence from longitudinal study of ageing in India

**DOI:** 10.1186/s12889-022-14697-2

**Published:** 2022-12-12

**Authors:** Rajeev Ranjan Singh, Priya Maurya

**Affiliations:** Department of Population and Development, International Institute for Population Sciences, Mumbai, 400088 Maharashtra India

**Keywords:** Falls, India, Injury, Older adults, Visual impairment

## Abstract

**Background:**

The present study determines the prevalence and correlates of falls, multiple falls, and injuries, focusing on visual impairment among the older adult and elderly population in India. Additionally, owing to the higher prevalence of falls and visual impairment among women, a sex-stratified analysis has also been done in the present study.

**Methods:**

The study utilized the data from the first wave of the Longitudinal Ageing Study in India (LASI wave-1), conducted during 2017-2018. Bivariate with chi-square and multivariate analyses were performed to fulfill the objective.

**Results:**

Around 34% of population had low vision (male:30% and female: 38%), while blindness prevalence was 1.63% (males: 1.88% and females: 1.41%). The fall was higher among females and increased across the gender with increasing visual impairment and blindness levels. The unadjusted odds of falls were 16% higher among individuals with low vision and 40% higher among individuals with blindness than with normal vision, and comparatively higher odds among females than males.

**Conclusion:**

In summary, falls and visual impairment is public health challenge and needs to be addressed. Visual impairment is preventable in most cases, so it may be a modifiable target for reducing the risk of falls.

**Supplementary Information:**

The online version contains supplementary material available at 10.1186/s12889-022-14697-2.

## Introduction

Population ageing is an inevitable and irreversible demographic reality resulting in a sustained change in the population's age structure, which is driven by increasing life expectancy and decreasing fertility. In India, the share of elderly population is predicted to be more than double from 9.4% in 2017 to 19.1% by 2050 [1]. The increasing older population has impacted all sectors of society, including the market, labor force, public health needs, and economic and social implications [2]. This population transition compelled to prepare for the economic and social shift with an ageing population to accomplish the pledge of the 2030 agenda of Sustainable Development Goal of “leaving no one left behind” [1]. At the biological level, ageing is a sequential or progressive change resulting from deteriorating the physical functions necessary for survival and reproduction. It increases the risk of debility, disease, and death [3]. The most common concern in old age includes hearing loss, cataracts, arthritis, heart disease, respiratory disease, alzheimer’s disease, osteoporosis, diabetes, falls, depression, and oral health [4, 5].

Falls are a substantial cause of morbidity, disability, and mortality across all age groups. It is defined as an event resulting in a person returning to rest inadvertently on the ground or at a lower level than the previous one [6, 7]. Globally, fall is the second leading cause of unintentional death after road traffic injuries, higher among older adults [5]. Falls are associated with increased injuries-related hospitalization and a decline in health-related quality of life [8]. It is manifested that about one-third of older adults aged 65 years and above report a fall at least once every year, and half of the cases are recurrent [9]. Evidence suggests that some people develop a fear of falling ensuing a fall, resulting in a decrease in physical activities as they attempt to avoid recurrent falls [10]. The morbidity experienced by falls further leads to comorbid conditions such as osteoporosis, osteopenia, and other chronic conditions that put substantial costs on the health system [8]. The prevalence of falls ranges from 14% to 53% among older Indian adults [11]. Therefore, it is essential to draw attention to falls along with the increasing older population.

Previous literature identified a wide range of risk factors for falls and related injuries among older adults, such as musculoskeletal, vestibular, somatosensory, and visual impairment [11–13]. Visual impairment is related to reducing one's ability to detect hazards that lead to loss of confidence and independence, poor subjective well-being, low level of social interaction, fear of falling, and risk of falling [14, 15]. In 2020, 1.1 billion people had distance vision impairment or uncorrected presbyopia worldwide, which will increase to 1.8 billion by 2050 [16]. The loss of visual function among older people can be directly attributed to anatomical changes in eyeball and functional decline. The anatomical changes bring down the quality of sensory inputs to high-level visual processing due to age, contributing to declining visual function performance [13]. Visual impairment is highly prevalent among older adults and a frequent risk factor for fall incidence [17, 18]. Cross-sectional studies show that those with visual impairment had a prevalence of falls ranging from 26% to 38% [15, 19]. Compared to those without vision loss, individuals with low vision have two times higher chance of falls, multiple falls, and related fractures [20]. Falls among visually impaired people can occur due to intrinsic and extrinsic factors [15]. In developing countries like India, people with visual impairment face different challenges, such as accessibility, navigator barriers, poverty, and healthcare utility challenges [21, 22].

Though population-based studies from states of India reported a higher prevalence of falls, recurrent falls, and related injuries among visually impaired elderly [19, 23], the knowledge about falls, multiple falls, and related injuries associated with visual impairment at the national level is sparse. In light of the greying population in India, we believe more attention is needed to the role of vision and visual impairment to minimize the risk of falls, multiple falls, and fall-related injuries among older adults. The present study determines the prevalence and correlates of falls, multiple falls, and injuries with a special focus on visual impairment among the older adult population in India. Additionally, owing to the higher prevalence of falls and visual impairment among women, a sex-stratified analysis has also been done in the present study.

## Material and methods

### Data

The study utilized the data from the first wave of the Longitudinal Ageing Study in India (LASI wave-1) which was conducted during 2017-2018. LASI is a nationally representative survey conducted by the International Institute for Population Sciences, Mumbai, in collaboration with Harvard T. H. Chan School of Public Health (HSPH) and the University of Southern California (USC) under the supervision of the Ministry of Health and Family Welfare (MoHFW), Government of India [24]. The survey covered 72,250 individuals aged 45 and above and their spouses, irrespective of age, across all the states and union territories in India. The major focus of the survey was to capture the health and socio-economic aspects of population ageing in India. LASI also includes an individual module on biomarkers and direct health examination of individuals. The survey conducting agencies obtained prior informed consent from all the respondents. LASI utilized multistage stratified sampling to sort the required sampling unit. Within each state, a three-stage sampling design in rural areas and a four-stage sampling design in urban areas were adopted in LASI wave-1. In the first stage, in rural areas, the primary sampling unit (sub-districts; Tehsil/Taluka) was selected, followed by villages in the second stage. In the third and final stage, the household was selected to be surveyed. For urban areas similar to rural areas in the first stage, primary sampling unit, that is, sub-district (sub-districts; Tehsil/Taluka), was selected, followed by wards in the second stage, and in the third stage, census enumeration blocks (CEB) were selected. In the fourth and last stage, households were selected from the selected CEB. The detailed methodology of the survey design is available in the survey report [24]. The response rate was 92.7% at the household level and 95.6% at the individual level [24]. The effective sample size for the present study was 56,355 older adults and elderly aged 45 years and above.

### Variable description

#### Outcome variables

The primary outcome variable of the study was falls which was assessed through a direct question on self-reported falls at the time of survey: “In the past two years, have you fallen down?”. Individuals who responded ‘no’ were categorized as ‘no falls’, while those who answered in affirmation were classified as ‘having falls’. Individuals who reported in affirmation of falls further they were asked about the frequency of falls “How many times have you fallen in the last two years?” The response variable multiple falls was recoded as 'no’ if individuals had one falls otherwise, ‘yes’. Individuals with a falls history were asked about falls related injuries, and the response variable falls-injury was recoded as ‘no’ and ‘yes’.

#### Key explanatory variable

Visual impairment was a key explanatory variable in the study. To measure visual impairment, a medically trained enumerator included the participants to measure the near and distance vision (not blind by birth) for each eye with the best correction available, irrespective of using specs or lens; that is, those using specs/lens and have a normal vision then, they will be considered as having normal visual acuity. However, if the individual’s vision is not normal (in one eye or both eye) even after using glasses or lenses, they will be considered as visually impaired with those who are visually impaired and not using any specs/lens. The measurement was carried out with WHO suggested standard measure, using the computer-assisted personal interview (CAPI) based tumbling E log medicine administration record (MAR) chart or log mart vision chart [24]. The visual impairment was coded as “normal”, “low vision”, and “blindness”. The low vision was defined as either low near-vision or low-distance vision. The low-distance vision was defined as visual acuity equal to or poorer than 20/80 and/ or better than 20/200 in the better eye with the best correction available. Low near vision was defined as visual acuity equal to or poorer than 20/ 80 and equal to or better than 20/400 in the better eye with the best correction available. Blindness was defined when an individual could not detect light and count fingers at 2 feet, or visual acuity was poorer than 20/400 for near vision or poorer than 20/200 for distance vision in the better eye with the best correction available [24].

#### Other explanatory variables

Based on previous literature individual, health, and household level factors included in the present study.

#### Individual factors

Sex was coded as male and female. Age was categorized as 45-54, 55-64, 65-74, and 75+ years. Educational attainment was classified as no education, 1-5 years, 6-10 years, and more than 10 years. Marital status was recoded as currently married, widowed, and others (divorced/separated/deserted/live in relationship/never married). Living arrangement was categorized as living with spouse and children, living with children and others, living with spouse, and living alone. Working status was categorized as never worked, earlier worked, and currently working.

#### Health factors

Self-rated health was categorized as good (very good/good/fair) and poor (poor/very poor). To measure difficulty in the activity of daily living (ADL), respondents were asked about normal daily activities, “Do you have any difficulties in dressing, walking, bathing, eating, mobility, and going toilet?” A composite index was prepared from the above-mentioned questions. The response variable “difficulty in ADL” were coded 0 score as ‘no difficulty’ and 1 score as ‘having difficulty.’ The Cronbach’s alpha value for the ADL scale was 0.821. To measure the difficulty in instrumental activity of daily living (IADL), respondents were asked about such activities which let individuals live independently, “have you any difficulties in preparing a meal, shopping, making telephone, medication, doing work in garden or home, money handling and getting around?” A composite index was prepared from the above-mentioned questions. The response variable “difficulty in IADL” were coded 0 score as ‘no difficulty’ and 1 score as ‘having difficulty.’ The Cronbach’s alpha value for the IADL scale was 0.846. Sleep problem was categorized as no, rarely, occasionally, and frequently.

#### Household factors

The monthly per capita expenditure (MPCE) quintile was categorized from poorest to richest. Social hierarchy of people, that is, social caste, was categorized as scheduled tribe [ST], scheduled caste [SC], other backward class [OBC], and other castes. Religion was categorized as Hindu, Muslim, and others. Place of residence as rural and urban and regional divide was recoded as north, central, east, northwest, west, and south.

#### Statistical approach

The analysis was done for the older adults and elderly, that is, 45 and above age, excluding their younger spouses. For preliminary analysis, descriptive statistics and bivariate analysis were used. The Chi-square test was used to check the association and report the significance level. Further, binary logistic regression was carried out to evaluate the covariates of falls among older adults and elderly. Unadjusted (model 1) and adjusted with other independent variables (model 2) were applied. The results were presented as an odds ratio (OR) with 95% of confidence interval (CI).

## Results

Table 1 presents the sample characteristics of the study population, which consist of 54% of females and 46% of males. The number of females (64%) with no education was around two-fold higher than the males (32%). Regarding marital status, a higher proportion of older adults and elderly males (88%) were in the union, whereas the same prevalence for females was 64%. Around half of the respondents (48%) were currently working, which was higher among males (68.13%) than females (31.44%). About 18% of the respondents reported their health as poor, 15% had difficulties in ADL, and more than one-third had difficulties in IADL. Around 28% of the population belonged to lower social strata: scheduled tribe (8.62%) and scheduled caste (19.58%).

**Table 1:** Socio-economic and health profile of study participants n(%)

**Background characteristics**	**Total**	**Male**	**Female**
**Age (in years)**			
45-54	21613 (36.4)	9677 (35.08)	11936 (37.52)
55-64	17695 (30.8)	7940 (30.29)	9755 (31.24)
65-74	12168 (23.38)	6098 (24.3)	6070 (22.59)
75+	4879 (9.42)	2455 (10.33)	2424 (8.65)
**Educational attainment (in years)**			
No	25956 (49.82)	7913 (32.88)	18043 (64.28)
1-5	10538 (17.7)	5585 (21.36)	4953 (14.57)
6-10	13924 (21.65)	8530 (29.27)	5394 (15.14)
More than 10 years	5937 (10.84)	4142 (16.49)	1795 (6.01)
**Marital status**			
Currently married	42741 (74.93)	23137 (87.74)	19604 (64)
Widowed	11813 (22.25)	2244 (9.63)	9569 (33.02)
Others	1801 (2.82)	789 (2.63)	1012 (2.98)
**Living arrangement**			
With spouse and children	33351 (57.73)	18422 (69.16)	14929 (47.97)
With children and others	12446 (22.53)	2852 (11.32)	9594 (32.1)
With spouse	8619 (16.2)	4408 (17.89)	4211 (14.77)
Living alone	1939 (3.54)	488 (1.64)	1451 (5.16)
**Working status**			
Never worked	15419 (25.97)	1010 (2.87)	14409 (45.7)
Earlier worked	14165 (25.69)	7675 (29)	6490 (22.87)
Currently working	26771 (48.34)	17485 (68.13)	9286 (31.44)
**Self-rated health**			
Good	47157 (82.61)	22362 (84.08)	24795 (81.36)
Poor	9198 (17.39)	3808 (15.92)	5390 (18.64)
**ADL difficulty**			
No	48947 (84.9)	23305 (86.84)	25642 (83.24)
Yes	7408 (15.1)	2865 (13.16)	4543 (16.76)
**IADL difficulty**			
No	38650 (64.52)	20189 (73.89)	18461 (56.52)
Yes	17705 (35.48)	5981 (26.11)	11724 (43.48)
**Sleep Problem**			
No	34298 (60.76)	16885 (66.58)	17413 (55.8)
Rarely	12402 (21.3)	7309 (18.84)	5093 (23.41)
Occasionally	6446 (11.75)	4028 (9.39)	2418 (13.77)
Frequently	3209 (6.19)	1963 (5.2)	1246 (7.03)
**MPCE quintile**			
Poorest	11024 (20.93)	5080 (20.66)	5944 (21.15)
Poorer	11371 (21.28)	5264 (21.27)	6107 (21.3)
Middle	11383 (20.52)	5291 (20.61)	6092 (20.44)
Richer	11385 (19.58)	5334 (19.49)	6051 (19.66)
Richest	11192 (17.69)	5201 (17.97)	5991 (17.45)
**Caste**			
Others	15813 (26.47)	7350 (26.57)	8463 (26.38)
OBC	21186 (45.67)	9861 (45.72)	11325 (45.62)
SC	9381 (19.25)	4328 (19.18)	5053 (19.3)
ST	9975 (8.62)	4631 (8.52)	5344 (8.7)
**Religion**			
Hindu	41221 (82.5)	19224 (82.95)	21997 (82.11)
Muslim	6736 (11.09)	3040 (11.01)	3696 (11.15)
Others	8398 (6.41)	3906 (6.04)	4492 (6.74)
**Place of residence**			
Urban	19663 (30.44)	8950 (29.12)	10713 (31.56)
Rural	36692 (69.56)	17220 (70.88)	19472 (68.44)
**Region**			
North	10319 (12.55)	4761 (12.29)	5558 (12.77)
Central	7567 (20.61)	3684 (21.99)	3883 (19.42)
East	10247 (24.13)	4846 (25.06)	5401 (23.34)
Northeast	7524 (3.55)	3623 (3.68)	3901 (3.44)
West	7339 (15.73)	3355 (15.13)	3984 (16.24)
South	13359 (23.43)	5901 (21.85)	7458 (24.78)
Total	**56,355 (100)**	**26,170 (46.06)**	**30,185 (53.94)**

Table 2 presents the measured visual impairment and falls-related characteristics of the older adults and elderly population in India. Around 34% of the total population had low vision, which was higher among females (38%) compared to males (30%), while blindness was higher among males (1.88%) than females (1.41%). Fall was around 20% among older adults and elderly, with a higher proportion among females (21%). In case of multiple falls, the total proportion increased to 40% again, with higher among females (42%) than males (37%). The proportion of falls leading to any injury was more than 50% among both males and females.

**Table 2:** Measured visual impairment and falls specific characteristics of the study population

**Variables**	**Total**		**Male**		**Female**	
	**n (%)**	**95% CI**	**n (%)**	**95% CI**	**n (%)**	**95% CI**
**Visual impairment**						
Normal	36,446 (64.07)	(63.24 - 64.89)	18,196 (68.25)	(67.1 - 69.37)	18,250 (60.50)	(59.29 - 0.62)
Low vision	19,176 (34.31)	(33.5 - 0.35)	7,605 (29.87)	(28.78 - 30.99)	11,571 (38.09)	(36.91 - 0.39)
Blindness	733 (1.63)	(1.44 - 0.02)	369 (1.88)	(1.55 - 2.27)	364 (1.41)	(1.21 - 0.02)
**Fall**						
No	46,344 (80.15)	(79.45 - 80.84)	22,137 (82.48)	(81.63 - 83.3)	24,207 (78.08)	(77.08 - 0.79)
Yes	10,011 (19.85)	(19.16 - 0.21)	4,033 (17.52)	(16.7 - 18.37)	5,978 (20.78)	(20.78 - 0.23)
**Multiple fall**^a^						
No	5,997 (59.72)	(57.67 - 61.74)	2,533 (62.75)	(60.34 - 65.11)	3,464 (57.65)	(54.67 - 0.61)
Yes	4,004 (40.28)	(38.26 - 0.42)	1,497 (37.25)	(34.89 - 39.66)	2,507 (42.35)	(39.44 - 0.45)
**Fall Injury**^a^						
No	4,815 (44.68)	(42.84 - 46.53)	1,920 (43.93)	(41.51 - 46.39)	2,895 (45.19)	(42.57 - 0.48)
Yes	5,210 (55.32)	(53.47 - 0.57)	2,121 (56.07)	(53.61 - 58.49)	3,089 (54.81)	(52.16 - 0.57)

^a^Sample varies due to missing value

**Fig. 1:**
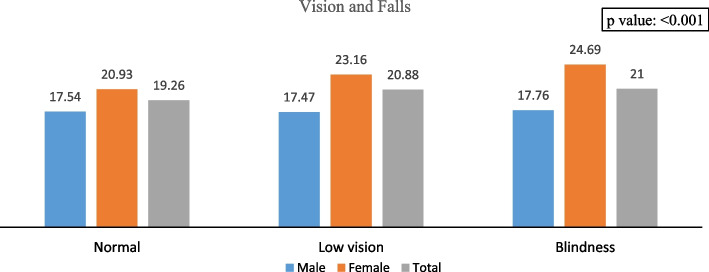
Association between measured visual impairment and fall among older adults and elderly in India, 2017-18

**Fig. 2:**
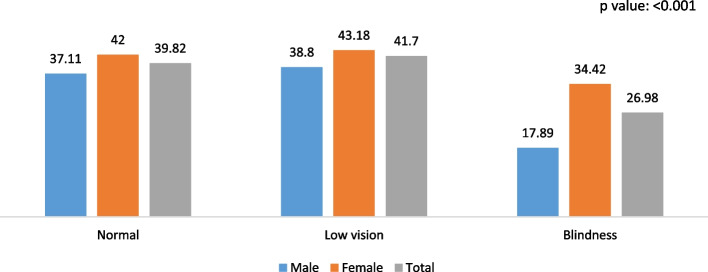
Association between measured visual impairment and multiple fall among older adults and elderly in India, 2017-18

**Fig. 3:**
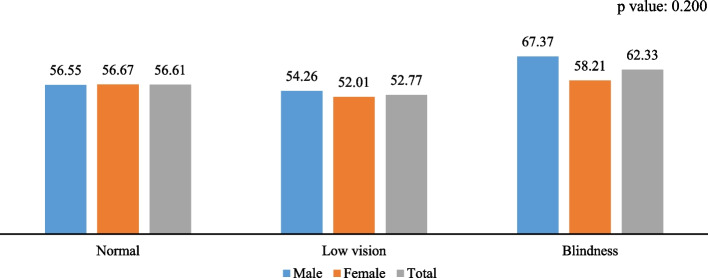
Association between measured visual impairment and injury among older adults and elderly in India, 2017-18

Figures 1, 2, and 3 depict the gender-specific measured visual impairment and fall, multiple falls, and fall-related injury among older adults and elderly. The fall was higher among females and increased across the gender with increasing visual impairment and blindness levels. Similarly, the frequency of falls, i.e., multiple falls and injury caused by falls, were higher among individuals with low vision and blindness. Specifically, falls were 19% among individuals with normal vision and 21% among those with low vision and blindness.

Table 3 presents the prevalence estimates of falls among older adults and elderly in India by background characteristics. All the selected background variables showed a significant association with falls. Overall, fall was higher among females (22%) than males (18%). The analysis showed a positive association of falls with increasing age, MPCE quintile, rural residents, low level of education, poor self-rated health, and older adults and elderly with sleep problems. In the case of marital status, the fall was higher among widowed (24%) compared to those currently married (19%) or others (17%). Similarly, with the living arrangements, the fall was higher among those living alone (25%), with more than the double proportion among females (28%) to males (13%). Similar findings were observed for multiple falls and fall-related injuries presented in the supplementary table (Supplementary Tables 1 and 2).

**Table 3:** Prevalence estimate of falls by background characteristics among older adults and elderly in India, 2017-2018

**Background characteristics**	**Total**	**Male**	**Female**	***p* value**
**Age (in years)**				<0.001
45-54	16.99	15.79	17.94	
55-64	20.31	17.64	22.52	
65-74	22.5	18.68	26.01	
75+	22.79	20.3	25.33	
**Educational attainment (in years)**				<0.001
No	21.04	18.59	22.12	
1-5	21.63	19.73	24	
6-10	18.94	17.04	22.08	
More than 10 years	13.25	13.38	12.95	
**Marital status**				<0.001
Currently married	18.63	17.32	20.17	
Widowed	24.32	19.4	25.55	
Others	16.73	17.31	16.28	
**Living arrangement**				<0.001
With spouse and children	18.39	17.17	19.9	
With children and others	23.47	19.55	24.65	
With spouse	18.97	18	19.97	
Living alone	24.46	12.89	27.6	
**Working status**				<0.001
Never worked	20.12	15.31	20.38	
Earlier worked	22.11	18.59	25.92	
Currently working	18.5	17.16	20.97	
**Self-rated health**				<0.001
Good	18.06	15.91	19.96	
Poor	28.34	26.04	30.02	
**ADL difficulty**				<0.001
No	18.18	16.48	19.69	
Yes	29.21	24.36	32.47	
**IADL difficulty**				<0.001
No	17.08	16.09	18.19	
Yes	24.87	21.56	26.57	
**Sleep Problem**				<0.001
No	16.38	15.05	17.73	
Rarely	22.68	20.34	24.28	
Occasionally	26.97	23.75	28.84	
Frequently	30.65	27.66	32.55	
**MPCE quintile**				<0.001
Poorest	18.31	16.23	20.05	
Poorer	19.88	19.08	20.57	
Middle	20.08	17.82	22.02	
Richer	19.82	17.56	21.73	
Richest	21.38	16.76	25.43	
**Caste**				<0.001
Others	20.52	17.36	23.25	
OBC	18.92	16.61	20.9	
SC	22.18	19.79	24.21	
ST	17.46	17.79	17.18	
**Religion**				<0.001
Hindu	19.9	17.78	21.72	
Muslim	19.35	15.21	22.84	
Others	20.06	18.14	21.53	
**Place of residence**				<0.001
Urban	16.23	13.16	18.65	
Rural	21.43	19.31	23.3	
**Region**				<0.001
North	15.45	13.72	16.87	
Central	19.91	17.34	22.4	
East	26.99	24.31	29.44	
Northeast	18.93	15.77	21.82	
West	20.48	17.45	22.89	
South	14.5	12.39	16.1	
**Total**	**19.85**	**17.52**	**21.83**	

Table 4 presents the logistic regression estimate of falls among older adults and elderly in India. The unadjusted odds of fall were 1.16 [UOR: 1.16, 95% CI: 1.11-1.21] times higher among individuals with low vision and 1.40 [UOR: 1.40, 95% CI: 1.17-1.67] times higher among blind than with normal vision and comparatively higher odds among females compared to male. Falls has a significant association in the case of adjusted odds ratio with increasing age, education, poor self-rated health, difficulties in active daily living, difficulties in the instrument of activity of daily life, sleep problem, higher MPCE quintile, individuals with lower strata of the caste system, rural dwellers, and females. The odds of falls were 1.53 [AOR: 1.53, 95% CI: 1.44-1.162] times higher among individuals who rated their health as poor compared to good. For those having difficulties in ADL, the odds were 1.40 [AOR: 1.40, 95% CI: 1.31-1.49] times higher compared to those who don’t. Those with frequent sleep problems were 1.79 [AOR: 1.79, 95% CI: 1.64-1.95] times more likely to fall than those without sleep problems. The odds of falls were 1.23 [AOR: 1.23, 95% CI: 1.16-1.29] times higher among rural residents than that of urban, and the overall odds of falls were 1.30 [AOR: 1.30, 95% CI: 1.23-1.38] times higher among female to the male (Table 4). Further, people with low vision were more likely to have multiple falls in the unadjusted model, whereas blindness did not show a significant association with multiple falls (Supplementary Table 3). Additionally, the association between fall-related injury and visual impairment was insignificant in both unadjusted and unadjusted models (Supplementary Table 4).

**Table 4:** Logistic regression estimate of falls among older adults and elderly in India, 2017-2018

**Variables**	**Total**		**Male**		**Female**	
	**UOR (95% CI)**	**AOR (95% CI)**	**UOR (95% CI)**	**AOR (95% CI)**	**UOR (95% CI)**	**AOR (95% CI)**
**Visual impairment**						
Normal						
Low vision	1.16***(1.11 - 1.21)	1.01 (0.96 - 1.06)	1.09*(1.02 - 1.18)	0.97 (0.89 - 1.04)	1.14***(1.08 - 1.21)	1.04 (0.98 - 1.11)
Blindness	1.4***(1.17 - 1.67)	1.13 (0.94 - 1.35)	1.28 (0.98 - 1.67)	1.01 (0.77 - 1.34)	1.52**(1.2 - 1.92)	1.24 (0.97 - 1.59)
**Age (in years)**						
45-54®						
55-64		1.1**(1.04 - 1.17)		1.09 (1 - 1.19)		1.12**(1.04 - 1.2)
65-74		1.11**(1.04 - 1.18)		1.02 (0.92 - 1.13)		1.19***(1.09 - 1.31)
75+		1.08 (0.98 - 1.19)		1.07 (0.92 - 1.23)		1.12 (0.99 - 1.26)
**Educational attainment (in years)**						
No®						
1-5		1.17***(1.1 - 1.25)		1.08 (0.98 - 1.19)		1.23***(1.14 - 1.34)
6-10		1 (0.93 - 1.06)		0.94 (0.85 - 1.03)		1.06 (0.96 - 1.16)
More than 10 years		0.8***(0.73 - 0.88)		0.77***(0.68 - 0.87)		0.83*(0.71 - 0.97)
**Marital status**						
Widowed®						
Currently married		1.08 (0.89 - 1.3)		0.59*(0.38 - 0.9)		1.44**(1.15 - 1.8)
Others		0.84*(0.73 - 0.97)		0.81 (0.63 - 1.04)		0.89 (0.74 - 1.06)
**Living arrangement**						
With spouse and children®						
With children and others		1.16 (0.96 - 1.4)		0.63*(0.42 - 0.96)		1.53***(1.23 - 1.9)
With spouse		0.9**(0.85 - 0.97)		0.91 (0.83 - 1.01)		0.9*(0.82 - 0.99)
Living alone		1.07 (0.86 - 1.32)		0.44***(0.27 - 0.69)		1.53**(1.19 - 1.96)
**Work status**						
Earlier worked®						
Never worked		0.89**(0.83 - 0.95)		0.94 (0.77 - 1.14)		0.82***(0.76 - 0.88)
Currently working		1.05 (0.99 - 1.11)		1.12*(1.02 - 1.23)		0.96 (0.88 - 1.05)
**Self-rated health**						
Good®						
Poor		1.53***(1.44 - 1.62)		1.56***(1.43 - 1.71)		1.51***(1.4 - 1.62)
**ADL difficulty**						
No®						
Yes		1.4***(1.31 - 1.49)		1.39***(1.25 - 1.55)		1.39***(1.28 - 1.51)
**IADL difficulty**						
No®						
Yes		1.2***(1.14 - 1.27)		1.27***(1.16 - 1.38)		1.17***(1.09 - 1.25)
**Sleep Problem**						
No®						
Rarely		1.3***(1.23 - 1.37)		1.2***(1.1 - 1.31)		1.37***(1.27 - 1.47)
Occasionally		1.52***(1.42 - 1.62)		1.4***(1.25 - 1.56)		1.6***(1.47 - 1.74)
Frequently		1.79***(1.64 - 1.95)		1.65***(1.43 - 1.9)		1.87***(1.67 - 2.08)
**MPCE quintile**						
Poorest®						
Poorer		1.13**(1.06 - 1.22)		1.17**(1.05 - 1.31)		1.11*(1.01 - 1.22)
Middle		1.23***(1.14 - 1.32)		1.2**(1.07 - 1.34)		1.26***(1.14 - 1.38)
Richer		1.31***(1.21 - 1.41)		1.29***(1.15 - 1.45)		1.32***(1.19 - 1.45)
Richest		1.43***(1.33 - 1.55)		1.44***(1.28 - 1.62)		1.42***(1.29 - 1.58)
**Caste**						
Others®						
OBC		1.06 (1 - 1.12)		1.07 (0.98 - 1.18)		1.05 (0.97 - 1.13)
SC		1.11**(1.04 - 1.19)		1.15*(1.03 - 1.29)		1.08 (0.99 - 1.19)
ST		0.71***(0.65 - 0.77)		0.78***(0.68 - 0.88)		0.67***(0.6 - 0.75)
**Religion**						
Hindu®						
Muslim		0.87***(0.81 - 0.94)		0.74***(0.65 - 0.83)		0.98 (0.89 - 1.07)
Others		1.06 (0.98 - 1.15)		1.02 (0.91 - 1.16)		1.08 (0.98 - 1.2)
**Place of residence**						
Urban®						
Rural		1.23***(1.16 - 1.29)		1.28***(1.18 - 1.39)		1.19***(1.11 - 1.27)
**Region**						
North®						
Central		1.27***(1.17 - 1.38)		1.26***(1.11 - 1.43)		1.27***(1.14 - 1.42)
East		1.95***(1.81 - 2.1)		2.02***(1.8 - 2.26)		1.89***(1.72 - 2.08)
Northeast		0.89*(0.81 - 0.98)		0.92 (0.79 - 1.06)		0.85*(0.75 - 0.97)
West		1.17***(1.08 - 1.28)		1.23**(1.08 - 1.4)		1.12*(1 - 1.25)
South		0.85***(0.79 - 0.92)		0.9 (0.8 - 1.01)		0.81***(0.73 - 0.9)
**Gender**						
Male®						
Female		1.3***(1.23 - 1.38)				

Note: *AOR* Adjusted Odds Ratio, *UOR* Unadjusted Odds Ratio, *CI* Confidence Interval

^*^if *p* < 0.05, **if *p* < 0.01, ***if *p* < 0.001

## Discussion

Falls are the most common impaired health condition in old age, but it is not an inevitable part of growing old [9, 25]. Visual impairment is one of the major contributors to falling and fall-related injuries [13, 18]. However, limited evidence is ascribed to the association of falls and visual impairment among older Indian adults. Therefore, this study contributes important and novel epidemiological data on the prevalence of falls and their association with visual impairment after controlling various socio-demographic and behavioral factors among older adults in India. In this study of a nationally representative sample of Indian older adults aged 45 years and above, we found that falls, multiple falls, and fall-related injuries were significantly more prevalent among individuals with visual impairment. This implies that the burden of falls associated with visual impairment is prevailing and needs to be addressed.

Visual impairment is one of the most common morbidity among older adults, with the prevalence increasing in old age [26]. The present study found that more than one-third of the study participants (34.31%) had a low vision which was higher among females (38.09%) than males (29.8%), and 1.63% of the elderly had blindness. Our estimates are higher than the previous studies from India. For instance, the national blindness and visual impairment survey found that the prevalence of visual impairment (including blindness) was 13.76% among older adults aged 50 years and above [27]. Another survey on ocular morbidity from India reported that 30% of the study participants had visual impairment [19]. These variations might be attributed to the mean age of the study subjects, the definition of the outcome, and the sample population. Further, visual impairment is related to poor depth perception, which encompasses binocular stereopsis, or the ability to perceive objects in three dimensions, and monocular cues involving depth and motion cues resulting in a higher incidence of falls among visually impaired individuals [12, 13].

In this population-based study of older Indian adults, 19.85% of individuals reported falls in the last two years. Of them, 40.28% and more than half (55.32%) reported multiple falls and fall-related injuries. A systematic review from India mentioned that the prevalence of falls among older people aged 60 years and above ranged from 14% to 53% [11]. A similar study also stated that falls led to 20% to 30% of mild to severe injuries and 10% to 15% of all emergency visits for medical care. Although, Marmamula et al. (2020) found 7.26% prevalence of falls among elderly in the last two weeks of the survey, and half of them needed medical treatment for the same [19].

Furthermore, a study propounds the benefits of exercise and dry needling to improve the rate of falls among elderly patients with osteoarthritis [28]. In aliening with previous evidence from a systematic review [11], gender differences in the occurrence of falls and multiple falls were also observed in the present study, as women had a higher prevalence than men. The researchers explain this disparity through gender differences in physical activity levels, muscle weakness, and loss of lower body strength [29–31]. A systematic review focused on muscle strengthening exercise effectiveness in postmenopausal women with osteoporosis suggested that muscle strengthening exercise, along with other therapeutic modalities, improves other capacities such as muscle strength, balance, functionality, and quality of life that is key in the primary prevention of falls among older women [32].

The decline in visual functions leads to the problem with balance control, gait disorder, and reduced ability to perceive contrast, resulting in an increased risk of falls among older adults [6, 13]. Our findings substantiate previous investigations confirming that older adults with low visual impairment and blindness were significantly more likely to fall than their sighted peers, and this association was stronger among women. Previous studies from India, such as Beaver Dam Eye Study and the Blue Mountain Eye Study, also reported a significant positive association between falls and visual impairment [14, 33]. Recent evidence from the HOMES study in India also found that the elderly with low vision were 51% more likely to fall [19]. A longitudinal study over five years by Hong et al. (2014) found that those with unilateral visual impairment had 27% higher chances of fractures among elderly than those who have normal vision [18]. However, Wood et al. (2011) found in their study that visual acuity was related to a rise in elevated risk of falls but not to an increased risk of falls-related injuries [34]. Evidence from literature suggested that falls were more prevalent among older people, and those with reduced visual acuity were 1.7 times and 1.9 times more likely to have falls and multiple falls compared to fully sighted populations, respectively [20]. A recent cohort study suggested that performing multicomponent exercise rehabilitation treatment will be favourable to improving the rate of falls and frailty in older adults [32]. Interestingly, the current study also observed that older adults with blindness were less likely to have multiple falls than their counterparts. This could be because the person with blindness and a history of falls develops fear of falling and reduces their physical activity and, therefore, might also reduce the chance of falls [10].

Our study pointed out that maximum falls, multiple falls, and injuries were present among the oldest-old age group, as reflected in multivariate analysis, which might be the result of age-related degeneration in sensory organs [35]. Females, illiterate, widowed, individuals who never worked, belonging to the poorest wealth quintile, and individuals residing in rural areas were more vulnerable to falls in our study, which is consistent with previous evidence [19]. For females, the possible explanation could be that they generally do daily household chores [36], which require higher movement vis-a-vis lower level of employment [37], resulting in gradual degradation in visual acuity, which leads to multiple falls and injuries among elderly. The other might have financial constraints in accessing health services, lack of awareness due to the lower educational attainment, and inaccessibility of resources that hold them back from accessing visual acuity services [38, 39]. In affirmation with previous evidence [19, 40], our findings also confirmed that poor health status is a risk factor for visual impairment among older adults. The higher prevalence of the chronic disease among the elderly also leads to vision loss [40], specially diabetes; globally, around 3.9 million people have diabetic retinopathy [41]. The fall among the elderly is related to sleep quality as well, elderly with frequent sleep problems have a higher probability of falls and fall-related injuries [42]. In line with earlier studies, elderly with low self-rated health and difficulties in performing ADL and IADL have a higher probability of falls [43, 44].

This study has limitations too. First, the cross-sectional nature of data does not allow for establishing the causal association between the variables. Secondly, the outcome variable, i.e., falls, multiple falls, and injuries, were self-reported, which caused reporting bias. Thirdly, the study did not include other potential factors that affect falls, multiple falls, and injuries, such as cognition and depth balance.

## Conclusion

In summary, falls and visual impairment is public health challenges and must be addressed. The findings of the present study strengthen the evidence that there is an independent relationship between falls, multiple falls and visual impairment, mainly among older adults with low vision. Visual impairment is often preventable, so it may be the modifiable target for reducing the risk of falls and related injuries. The finding suggests that simple and cost-effective strategies such as routine screening of vision loss and updating spectacles may be substantial for fall prevention among older adults.

## Supplementary Information

**Additional file 1:** **Supplementary Table 1.** Prevalence estimate of multiple falls among older adults and elderly in India, 2017-2018. **Supplementary Table 2.** Prevalence estimate of falls related injuries among older adults and elderly in India, 2017-2018. **Supplementary Table 3**. Logistic regression estimate of multiple falls among older adults and elderly in India, 2017-2018. **Supplementary Table 4**. Logistic regression estimate of fall related injuries among older adults and elderly in India, 2017-2018.

## Data Availability

Data for this study were extracted from the first wave of the Longitudinal Aging Study in India (2017-19) that is freely available in public domain on request through https://iipsindia.ac.in/sites/default/files/LASI_DataRequestForm_0.pdf.
