# Supplementary material for: Visual impairment and falls among older adults and elderly: evidence from longitudinal study of ageing in India
Source: BMC Public Health. 2022 Dec 12;22:2324. doi: 10.1186/s12889-022-14697-2 (PMC9746100; doi:10.1186/s12889-022-14697-2)
Supplement: Supplementary file 1 — Additional file 1: Supplementary Table 1. Prevalence estimate of multiple falls among older adults and elderly in India, 2017-2018. Supplementary Table 2. Prevalence estimate of falls related injuries among older adults and elderly in India, 2017-2018. Supplementary Table 3. Logistic regression estimate of multiple falls among older adults and elderly in India, 2017-2018. Supplementary Table 4. Logistic regression estimate of fall related injuries among older adults and elderly in India, 2017-2018. [file 12889_2022_14697_MOESM1_ESM.docx]

**Supplementary Table 1:** Prevalence estimate of multiple falls among older adults and elderly in India, 2017-2018.

| **Background characteristics** | **Total** | **Male** | **Female** | **p value** |
| --- | --- | --- | --- | --- |
| **Age (in years)** |  |  |  | <0.001 |
| 45-54 | 36.45 | 33.05 | 38.84 |  |
| 55-64 | 38.09 | 35.86 | 39.55 |  |
| 65-74 | 45.59 | 41.28 | 48.43 |  |
| 75+ | 44.66 | 43.15 | 45.9 |  |
| **Educational attainment (in years)** |  |  |  | <0.001 |
| No | 41.09 | 38.91 | 41.89 |  |
| 1-5 | 39.42 | 38.1 | 40.78 |  |
| 6-10 | 42.29 | 37.11 | 48.88 |  |
| More than 10 years | 30.76 | 31.24 | 29.61 |  |
| **Marital status** |  |  |  | <0.001 |
| Currently married | 38.05 | 36.33 | 39.78 |  |
| Widowed | 45.74 | 41.82 | 46.48 |  |
| Others | 43.61 | 48.92 | 39.36 |  |
| **Living arrangement** |  |  |  | <0.001 |
| With spouse and children | 37.51 | 36.26 | 38.83 |  |
| With children and others | 45.63 | 42.63 | 46.35 |  |
| With spouse | 39.34 | 36.59 | 41.91 |  |
| Living alone | 44.92 | 46.26 | 44.75 |  |
| **Working status** |  |  |  | <0.001 |
| Never worked | 42.15 | 27.7 | 42.74 |  |
| Earlier worked | 43.4 | 41.48 | 44.9 |  |
| Currently working | 37.2 | 35.66 | 39.53 |  |
| **Self-rated health** |  |  |  | <0.001 |
| Good | 38.02 | 34.36 | 40.59 |  |
| Poor | 47.13 | 46.57 | 47.48 |  |
| **ADL difficulty** |  |  |  | <0.001 |
| No | 38.73 | 36.29 | 40.55 |  |
| Yes | 45.7 | 41.56 | 47.77 |  |
| **IADL difficulty** |  |  |  | <0.001 |
| No | 37.46 | 36.89 | 38.02 |  |
| Yes | 43.79 | 37.99 | 46.2 |  |
| **Sleep Problem** |  |  |  | <0.001 |
| No | 36.5 | 33.1 | 39.44 |  |
| Rarely | 45.43 | 45.59 | 45.34 |  |
| Occasionally | 43.94 | 43.32 | 44.24 |  |
| Frequently | 40.89 | 34.49 | 44.26 |  |
| **MPCE quintile** |  |  |  | <0.556 |
| Poorest | 40.27 | 38.31 | 41.6 |  |
| Poorer | 40.36 | 38.2 | 42.08 |  |
| Middle | 38.61 | 34.76 | 41.3 |  |
| Richer | 40.95 | 40.47 | 41.28 |  |
| Richest | 41.32 | 34.12 | 45.46 |  |
| **Caste** |  |  |  | <0.091 |
| Others | 36.98 | 35.03 | 38.23 |  |
| OBC | 41.98 | 36.73 | 45.55 |  |
| SC | 42.22 | 41.42 | 42.77 |  |
| ST | 36.91 | 36.08 | 37.62 |  |
| **Religion** |  |  |  | <0.137 |
| Hindu | 40.39 | 36.81 | 42.92 |  |
| Muslim | 39.34 | 38.23 | 39.96 |  |
| Others | 40.35 | 41.53 | 39.59 |  |
| **Place of residence** |  |  |  | <0.111 |
| Urban | 40.95 | 35.87 | 43.76 |  |
| Rural | 40.06 | 37.63 | 41.84 |  |
| **Region** |  |  |  | <0.001 |
| North | 36.34 | 13.72 | 16.87 |  |
| Central | 42.11 | 17.34 | 22.4 |  |
| East | 42.43 | 24.31 | 29.44 |  |
| Northeast | 34.68 | 15.77 | 21.82 |  |
| West | 32.01 | 17.45 | 22.89 |  |
| South | 45.17 | 12.39 | 16.1 |  |
| **Total** | **40.28** | **37.25** | **42.35** |  |

**Supplementary Table 2:** Prevalence estimate of falls related injuries among older adults and elderly in India, 2017-2018.

| **Background characteristics** | **Total** | **Male** | **Female** | **p value** |
| --- | --- | --- | --- | --- |
| **Age (in years)** |  |  |  | <0.445 |
| 45-54 | 54.26 | 57.32 | 52.12 |  |
| 55-64 | 54.79 | 56.25 | 53.84 |  |
| 65-74 | 56.85 | 53.22 | 59.24 |  |
| 75+ | 56.18 | 58.46 | 54.33 |  |
| **Educational attainment (in years)** |  |  |  | <0.875 |
| No | 54.67 | 57.37 | 53.68 |  |
| 1-5 | 54.05 | 53.65 | 54.46 |  |
| 6-10 | 59.04 | 55.74 | 63.23 |  |
| More than 10 years | 52.88 | 57.83 | 40.85 |  |
| **Marital status** |  |  |  | <0.313 |
| Currently married | 55.69 | 56.82 | 54.56 |  |
| Widowed | 55.04 | 53.29 | 55.38 |  |
| Others | 47.48 | 42.31 | 51.6 |  |
| **Living arrangement** |  |  |  | <0.788 |
| With spouse and children | 55.95 | 56.94 | 54.9 |  |
| With children and others | 54.99 | 49.85 | 56.22 |  |
| With spouse | 54.62 | 57.06 | 52.35 |  |
| Living alone | 52.12 | 57.13 | 51.49 |  |
| **Working status** |  |  |  | <0.945 |
| Never worked | 58.01 | 66.33 | 57.67 |  |
| Earlier worked | 52.47 | 53.3 | 51.82 |  |
| Currently working | 55.57 | 56.96 | 53.46 |  |
| **Self-rated health** |  |  |  | <0.005 |
| Good | 54.51 | 54.93 | 54.21 |  |
| Poor | 57.79 | 59.75 | 56.55 |  |
| **ADL difficulty** |  |  |  | <0.001 |
| No | 54.38 | 54.66 | 54.17 |  |
| Yes | 58.59 | 62.34 | 56.72 |  |
| **IADL difficulty** |  |  |  | <0.001 |
| No | 53.23 | 54.44 | 52.03 |  |
| Yes | 57.94 | 59.52 | 57.28 |  |
| **Sleep Problem** |  |  |  | <0.001 |
| No | 55.19 | 56.4 | 54.14 |  |
| Rarely | 55.04 | 55.08 | 55.02 |  |
| Occasionally | 55.07 | 54.26 | 55.45 |  |
| Frequently | 57.13 | 59.21 | 56.02 |  |
| **MPCE quintile** |  |  |  | <0.001 |
| Poorest | 50.76 | 53.75 | 48.75 |  |
| Poorer | 53.5 | 50.86 | 55.59 |  |
| Middle | 56.91 | 59.73 | 54.95 |  |
| Richer | 53.96 | 54.64 | 53.5 |  |
| Richest | 61.64 | 62.84 | 60.96 |  |
| **Caste** |  |  |  | <0.001 |
| Others | 58.81 | 58.57 | 58.96 |  |
| OBC | 54.39 | 55.63 | 53.54 |  |
| SC | 54.71 | 55.89 | 53.9 |  |
| ST | 49.83 | 51.14 | 48.71 |  |
| **Religion** |  |  |  | <0.001 |
| Hindu | 55.47 | 55.62 | 55.37 |  |
| Muslim | 58.53 | 60.73 | 57.29 |  |
| Others | 48.07 | 55 | 43.6 |  |
| **Place of residence** |  |  |  | <0.080 |
| Urban | 55.61 | 58.24 | 54.14 |  |
| Rural | 55.23 | 55.46 | 55.06 |  |
| **Region** |  |  |  | <0.001 |
| North | 54.44 | 13.72 | 16.87 |  |
| Central | 56.85 | 17.34 | 22.4 |  |
| East | 55.95 | 24.31 | 29.44 |  |
| Northeast | 55.76 | 15.77 | 21.82 |  |
| West | 60.29 | 17.45 | 22.89 |  |
| South | 47.93 | 12.39 | 16.1 |  |
| **Total** | 55.32 | 17.52 | 21.83 |  |

**Supplementary Table 3**: Logistic regression estimate of multiple falls among older adults and elderly in India, 2017-2018.

| **Variables** | **Total** | | **Male** | | **Female** | |
| --- | --- | --- | --- | --- | --- | --- |
|  | **UOR (95% CI)** | **AOR (95% CI)** | **UOR (95% CI)** | **AOR (95% CI)** | **UOR (95% CI)** | **AOR (95% CI)** |
| **Visual impairment** |  |  |  |  |  |  |
| Normal |  |  |  |  |  |  |
| Low vision | 1.16***(1.07 - 1.26) | 1.05 (0.96 - 1.15) | 1.17*(1.02 - 1.35) | 1.03 (0.89 - 1.2) | 1.12*(1.01 - 1.24) | 1.06 (0.95 - 1.18) |
| Blindness | 0.85 (0.61 - 1.18) | 0.72 (0.51 - 1) | 0.59 (0.34 - 1.02) | 0.53*(0.3 - 0.93) | 1.05 (0.7 - 1.59) | 0.88 (0.58 - 1.35) |

Note: *if p < 0.05, **if p < 0.01, ***if p < 0.001; AOR: Adjusted Odds Ratio; UOR: Unadjusted Odds Ratio; CI: Confidence Interval. Adjusted with all the covariate considered in the study.

**Supplementary Table 4**: Logistic regression estimate of fall related injuries among older adults and elderly in India, 2017-2018.

| **Variables** | **Total** | | **Male** | | **Female** | |
| --- | --- | --- | --- | --- | --- | --- |
|  | **UOR (95% CI)** | **AOR (95% CI)** | **UOR (95% CI)** | **AOR (95% CI)** | **UOR (95% CI)** | **AOR (95% CI)** |
| **Visual impairment** |  |  |  |  |  |  |
| Normal |  |  |  |  |  |  |
| Low vision | 1.01 (0.93 - 1.09) | 1.01 (0.92 - 1.1) | 0.98 (0.86 - 1.12) | 1.02 (0.88 - 1.18) | 1.03 (0.93 - 1.14) | 0.99 (0.89 - 1.11) |
| Blindness | 1.33 (0.97 - 1.83) | 1.22 (0.88 - 1.69) | 1.37 (0.84 - 2.25) | 1.33 (0.8 - 2.21) | 1.31 (0.87 - 1.98) | 1.15 (0.75 - 1.76) |

Note: *if p < 0.05, **if p < 0.01, ***if p < 0.001; AOR: Adjusted Odds Ratio; UOR: Unadjusted Odds Ratio; CI: Confidence Interval. Adjusted with all the covariate considered in the study.
